# Exposure to Air Pollution, Genetic Susceptibility, and Psoriasis Risk in the UK

**DOI:** 10.1001/jamanetworkopen.2024.21665

**Published:** 2024-07-16

**Authors:** Junhui Wu, Yudiyang Ma, Jian Yang, Yaohua Tian

**Affiliations:** 1 School of Nursing, Peking University, Beijing, China; 2 Ministry of Education Key Laboratory of Environment and Health, and State Key Laboratory of Environmental Health (Incubating), School of Public Health, Tongji Medical College, Huazhong University of Science and Technology, Wuhan, China; 3 Department of Cardiology, The First College of Clinical Medical Science, China Three Gorges University & Yichang Central People’s Hospital, Yichang, China; 4 Hubei Key Laboratory of Ischemic Cardiovascular Disease, Yichang, China; 5 Hubei Provincial Clinical Research Center for Ischemic Cardiovascular Disease, Yichang, China

## Abstract

**Importance:**

Psoriasis is a common autoinflammatory disease influenced by complex interactions between environmental and genetic factors. The influence of long-term air pollution exposure on psoriasis remains underexplored.

**Objective:**

To examine the association between long-term exposure to air pollution and psoriasis and the interaction between air pollution and genetic susceptibility for incident psoriasis.

**Design, Setting, and Participants:**

This prospective cohort study used data from the UK Biobank. The analysis sample included individuals who were psoriasis free at baseline and had available data on air pollution exposure. Genetic analyses were restricted to White participants. Data were analyzed between November 1 and December 10, 2023.

**Exposures:**

Exposure to nitrogen dioxide (NO_2_), nitrogen oxides (NO_x_), fine particulate matter with a diameter less than 2.5 µm (PM_2.5_), and particulate matter with a diameter less than 10 µm (PM_10_) and genetic susceptibility for psoriasis.

**Main Outcomes and Measures:**

To ascertain the association of long-term exposure to NO_2_, NO_x_, PM_2.5_, and PM_10_ with the risk of psoriasis, a Cox proportional hazards model with time-varying air pollution exposure was used. Cox models were also used to explore the potential interplay between air pollutant exposure and genetic susceptibility for the risk of psoriasis incidence.

**Results:**

A total of 474 055 individuals were included, with a mean (SD) age of 56.54 (8.09) years and 257 686 (54.36%) female participants. There were 9186 participants (1.94%) identified as Asian or Asian British, 7542 (1.59%) as Black or Black British, and 446 637 (94.22%) as White European. During a median (IQR) follow-up of 11.91 (11.21-12.59) years, 4031 incident psoriasis events were recorded. There was a positive association between the risk of psoriasis and air pollutant exposure. For every IQR increase in PM_2.5_, PM_10_, NO_2_, and NO_x_, the hazard ratios (HRs) were 1.41 (95% CI, 1.35-1.46), 1.47 (95% CI, 1.41-1.52), 1.28 (95% CI, 1.23-1.33), and 1.19 (95% CI, 1.14-1.24), respectively. When comparing individuals in the lowest exposure quartile (Q1) with those in the highest exposure quartile (Q4), the multivariate-adjusted HRs were 2.01 (95% CI, 1.83-2.20) for PM_2.5_, 2.21 (95% CI, 2.02-2.43) for PM_10_, 1.64 (95% CI, 1.49-1.80) for NO_2_, and 1.34 (95% CI, 1.22-1.47) for NO_x_. Moreover, significant interactions between air pollution and genetic predisposition for incident psoriasis were observed. In the subset of 446 637 White individuals, the findings indicated a substantial risk of psoriasis development in participants exposed to the highest quartile of air pollution levels concomitant with high genetic risk compared with those in the lowest quartile of air pollution levels with low genetic risk (PM_2.5_: HR, 4.11; 95% CI, 3.46-4.90; PM_10_: HR, 4.29; 95% CI, 3.61-5.08; NO_2_: HR, 2.95; 95% CI, 2.49-3.50; NO_x_: HR, 2.44; 95% CI, 2.08-2.87).

**Conclusions and Relevance:**

In this prospective cohort study of the association between air pollution and psoriasis, long-term exposure to air pollution was associated with increased psoriasis risk. There was an interaction between air pollution and genetic susceptibility on psoriasis risk.

## Key Points

**Question:**

Is long-term exposure to air pollution associated with the development of psoriasis, and does genetic susceptibility interact with air pollution in the risk of psoriasis?

**Findings:**

In this cohort study with 474 055 participants, long-term exposure to air pollutants, including fine particulate matter with diameters less than 2.5 µm and less than 10 µm, nitrogen dioxide, and nitrogen oxides, was associated with an increased risk of psoriasis. Additionally, there was a significant interaction between air pollution and genetic predisposition for incident psoriasis.

**Meaning:**

In this study, long-term exposure to air pollution was associated with the development of psoriasis, and genetic susceptibility modified this association.

## Teaser

This cohort study examines whether long-term exposure to air pollution is associated with the risk of psoriasis and whether genetic susceptibility to psoriasis modifies this association.

## Introduction

As a common autoinflammatory disease, psoriasis manifests through various symptoms, including skin redness, discomfort, itching, and bleeding, potentially leading to physical disfigurement and impaired labor capability in affected individuals.^1,2,3,4^ Psoriasis has been linked to numerous cardiovascular diseases, arthritis, and even mortality.^4,5,6,7^ At present, there is no definitive cure for psoriasis. Existing treatments can only offer long-term control or provide a long period without flares, placing a substantial strain on health care systems and resulting in economic losses.^4,8^ Since the beginning of the 21st century, the incidence and prevalence of psoriasis have increased, especially in high-resource countries, posing a threat to human health.^9,10^ However, the etiology of psoriasis remains elusive, with the intricate interaction of genetic and environmental determinants presenting substantial hurdles to the effective prevention of this condition, thereby heightening public health concerns.

In light of the challenges posed by psoriasis, including its resistance to complete cure and the potential adverse effects of existing therapeutic interventions, it is imperative to discern the risk factors associated with this condition to facilitate early intervention.^4,11^ Air pollution is an important risk factor for the incidence of psoriasis. Earlier studies have found the association between short-term air pollution exposure and hospital visits for psoriasis in several countries, such as China,^12,13^ South Korea,^14^ and Italy.^15^ However, as psoriasis is a chronic autoinflammatory disease with a complex interaction between genetic and environmental factors, the existing studies on the associations of single air pollutants with psoriasis provide insufficient evidence for the association between air pollution and the incidence of psoriasis. Currently, there is a lack of long-term prospective studies assessing the impact of air pollution on the incidence of psoriasis as well as a shortage of research into the interaction between exposure to multiple air pollutants and genetic factors in relation to psoriasis risk.

Therefore, we aimed to conduct a prospective study of the association of long-term exposure to a range of air pollutants, including fine particulate matter with a diameter less than 2.5 μm (PM_2.5_), particulate matter with a diameter less than 10 μm (PM_10_), nitrogen dioxide (NO_2_), and nitrogen oxides (NO_X_), with incident psoriasis. This investigation is anchored in data from the UK Biobank database, which includes a recruited population of 500 000 participants.^16^ Using the extensive and high-quality genetic data on psoriasis within the UK Biobank,^17,18^ our investigation uses a polygenic risk score (PRS)^17,19^ that integrates multiple genetic variants to holistically identify individuals with elevated risk to explore the interaction of genetics and air pollution on psoriasis risk.

## Methods

### Research Design and Demographic Information

This study derives its population from the UK Biobank, a large-scale database established in the UK in 2006, encompassing more than 500 000 individuals aged 37 to 73 years.^16^ These participants have been under continuous longitudinal observation. A diverse range of information, including sociodemographic characteristics, physical examinations, medical history, biological samples, and imaging data, has been systematically collected via various methods, such as digital questionnaires, oral interviews, and clinical records.^20,21^ Ethical approval for the UK Biobank was obtained from the North West Multi-Centre Research Ethics Committee. Written informed consent was provided by each participant before the study, and researchers are allowed to use data from the UK Biobank without an additional ethical clearance. The analyses in this study were conducted under the UK Biobank application No. 69741. This cohort study followed the Strengthening the Reporting of Observational Studies in Epidemiology (STROBE) reporting guideline.

### Air Pollution Assessment

The Department for Environment, Food and Rural Affairs (DEFRA) in the UK actively compiles comprehensive data on near-surface air pollution,^22^ a resource that has significantly contributed to numerous studies.^23,24,25^ This open-source repository provided us with data on the annual average concentrations of various air pollutants from 2006 to 2021. This resource produces annual maps that detail the concentrations of air pollutants at a resolution of 1 × 1 km. These maps are the result of an air dispersion model that synthesizes data from the National Atmospheric Emissions Inventory, secondary inorganic aerosol measurements, and models for sources like dust resuspension. The estimated concentrations were further refined through calibration with measurements from background sites within DEFRA’s Automatic Urban and Rural Network. The agency conducts systematic comparisons of modeled and measured annual mean air pollutants to validate the model’s accuracy. These comparisons have consistently demonstrated a satisfactory correlation between the model and actual data. Details about the model’s performance are available online.^26^ Using methods similar to those in our and other studies,^27,28^ we calculated the exposure levels of PM_2.5_, PM_10_, NO_2_, and NO_x_ for each study participant based on their residential history as recorded by the UK Biobank.

### Genetic Information

The UK Biobank’s Research Access Platform supplied the standard PRS set, formulated for the UK Biobank cohort through a meta-analysis of various genome-wide association study (GWAS) sources. Previous research offers a more in-depth understanding of the PRS method and GWAS data used in the standard PRS set.^29^ Briefly, a standardized subgroup and uniform definitions for diseases and quantitative traits were used within the UK Biobank to evaluate the PRS consistently. The PRS algorithms were crafted through trait-specific meta-analyses, using a bayesian approach to integrate data across different ancestries and related traits. The PRS for each individual was calculated as the aggregate of per-variant posterior effect size, multiplied by the allele dosage throughout the genome. We acquired the PRS for psoriasis (Field identifier [ID], 26269) and categorized participants into 3 genetic risk levels: low (tertile 1), intermediate (tertile 2), and high (tertile 3).

### Evaluation of Outcomes

Incident psoriasis cases within the UK Biobank were identified through first occurrence records. These records were compiled by mapping data sources (Field IDs 131742 and 131743), which included primary care data, *International Classification of Disease *(*ICD*) codes from hospital inpatient data, *ICD* codes from death register records, and self-reported medical condition codes that were verified by nurses after a physician diagnosis. Further information is available online.^30^

### Ascertainment of Covariates

The covariates included in our study were primarily derived from the baseline questionnaire of the UK Biobank, which contains participants’ sociodemographic information, medical history, and physical examination data. Based on previous literature exploring factors related to psoriasis, we selected sociodemographic information (age, sex, ethnicity [classified as Asian or Asian British, Black or Black British, Chinese, mixed ethnicities, and White European], Townsend Deprivation Index [TDI], education, and employment status), health-related information (body mass index [BMI; calculated as weight in kilograms divided by height in meters squared], healthy diet score, alcohol consumption status, tobacco consumption status, and physical activity level), medical history (hypertension, diabetes, and hyperlipidemia), and kinship to other participants as covariates.^3,6,31^

### Analysis Population

Of the total population of 502 480 individuals, 10 582 were excluded due to baseline psoriasis, 1505 were excluded due to missing air pollution-related variables, and 16 338 were excluded due to genetic data loss. Finally, 474 055 individuals were included for analysis. We collected complete follow-up information from their entry into the study until the occurrence of psoriasis, death, or the end date of December 30, 2020. In analyses related to PRSs, considering population heterogeneity, we restricted the genetic analysis to 446 637 individuals with White European ethnicity.

### Statistical Analysis

In this study, we used time-varying Cox proportional hazards models to evaluate the associations among air pollutants (treated as a continuous variable or divided into quartile), genetic risk categories (utilizing 3 categories to describe the risk and low risk as the reference group), and the combined exposures of air pollutants and genetic risk on the incidence of psoriasis. The outcomes of these analyses were presented in the form of hazard ratios (HRs) and their respective 95% CIs. The validity of the proportional hazards assumption underpinning our models was evaluated using Schoenfeld residuals, with no deviations identified. We ensured the robustness of our models by adjusting for potential confounders. Furthermore, in the analysis of genetic data, adjustments were made for the genotyping batch and the initial 10 principal components. We also examined the persistence of associations at lower concentrations by including participants residing in areas where air pollutant levels fell below the prespecified thresholds based on the World Health Organization yearly air quality guidelines (10 μg/m^3^ for PM_2.5_, 20 μg/m^3^ for PM_10_, and 40 μg/m^3^ for NO_2_), as well as those living in areas below the median concentration of 23.9 μg/m^3^ for NO_x_.^32^ All analyses were conducted using R version 4.2.0 (R Project for Statistical Computing). Two-tailed *P* < .05 was considered significant.

For the presentation of continuous variables, we used the mean with SD, whereas categorical variables were expressed as frequencies with percentages. The Student *t* test, Mann-Whitney *U* test, or χ^2^ test were applied to facilitate comparisons of continuous or categorical variables across different groups. Variables with missing data (all instances being <5%) were treated as separate indicator categories.

The exploration of the dose-response association between air pollutant exposure and the risk of developing psoriasis was conducted using restricted cubic spline models. The determination of knots within these models was guided by the Akaike information criterion, and their placement was in accordance with Harrell’s recommendations.^33^ To investigate the effects of air pollution and genetic interactions on psoriasis risk, the model was assessed on an additive scale, leading to the calculation of the relative excess risk due to interaction and the attributable proportion.

A series of sensitivity analyses were conducted to bolster the validity of our findings. Sensitivity analyses involved excluding participants diagnosed with psoriasis within the initial 2 years of follow-up. Our analysis was further refined to only include participants who had resided at their current address for a duration exceeding 5 years. We calculated E-values to deal with unmeasured confounders, estimating the E-value as the minimum strength of association that unmeasured confounders should fully explain away a specific association between exposure and outcome of interest.^33^ A large E-value would support a reasonable inference concerning the unlikelihood of unmeasured confounders entirely explaining the observed associations between air pollutants and psoriasis incidence.^34^ To eliminate the effects of moving, we added a sensitivity analysis by restricting analyses to individuals who had the same address throughout the follow-up period. Moreover, we developed a directed acyclic graph (DAG) to determine whether potential covariates should be adjusted in the models. From the DAG (eFigure in Supplement 1), a minimally sufficient set of variables for adjustment were retained (age, education, employment, ethnicity, smoking, and TDI). We also performed a sensitivity analysis using these selected covariates.

## Results

The participant demographic characteristics at the start of the study are presented in Table 1. The study included 474 055 individuals with a mean (SD) age of 56.54 (8.09) years, 257 686 (54.36%) female participants, and 9186 (1.94%) Asian or Asian British, 7542 (1.59%) Black or Black British, and 446 637 (94.22%) White European participants. Over a median follow-up period of 11.91 years, 4031 new psoriasis cases were identified. Individuals with psoriasis exhibited elevated BMI, alongside increased rates of hypertension, hypercholesterolemia, and diabetes. Additionally, a greater percentage of these individuals were male and active smokers, whereas their engagement in physical activity was lower than those without incident psoriasis. For the distribution of air pollutants, refer to eTable 1 in Supplement 1. The mean (SD) yearly levels of PM_2.5_, PM_10_, NO_2_, and NO_X_ were recorded at 10.66 (2.27), 16.08 (3.06), 19.11 (6.46), 28.71 (12.11) μg/m^3^, respectively (eTable 3 in Supplement 1).

**Table 1.** Baseline Characteristics of Participants

Characteristics	Participants, No. (%)			*P* value
	Total (N = 474 055)	Without psoriasis (n = 470 024)	With psoriasis (n = 4031)	
Age, y				
<53	167 820 (35.40)	166 579 (35.44)	1241 (30.79)	<.001
53 to 63	148 478 (31.32)	147 193 (31.32)	1285 (31.88)	
>63	157 757 (33.28)	156 252 (33.24)	1505 (37.34)	
Sex				
Female	257 686 (54.36)	255 595 (54.38)	2091 (51.87)	.002
Male	216 369 (45.64)	214 429 (45.62)	1940 (48.13)	
Ethnicity				
White European	446 637 (94.22)	442 771 (94.20)	3866 (95.91)	<.001
Mixed	2769 (0.58)	2754 (0.59)	15 (0.37)	
Asian or Asian British	9186 (1.94)	9109 (1.94)	77 (1.91)	
Black or Black British	7542 (1.59)	7524 (1.60)	18 (0.45)	
Chinese	5691 (1.20)	5650 (1.20)	41 (1.02)	
Missing	2230 (0.47)	2216 (0.47)	14 (0.35)	
BMI				
<25.00	156 446 (33.00)	155 401 (33.06)	1045 (25.92)	<.001
25.00 to 28.63	159 754 (33.70)	158 455 (33.71)	1299 (32.23)	
>28.63	157 855 (33.30)	156 168 (33.23)	1687 (41.85)	
Employment				
Employed	271 465 (57.26)	269 392 (57.31)	2073 (51.43)	<.001
Retired	157 947 (33.32)	156 461 (33.29)	1486 (36.86)	
Unemployed	39 618 (8.36)	39 197 (8.34)	421 (10.44)	
Missing	5025 (1.06)	4974 (1.06)	51 (1.27)	
Education				
College or university degree	177 717 (37.49)	176 315 (37.51)	1402 (34.78)	<.001
Nondegree level	209 504 (44.19)	207 852 (44.22)	1652 (40.98)	
Missing	86 834 (18.32)	85 857 (18.27)	977 (24.24)	
Alcohol consumption status				
Never	20 950 (4.42)	20 792 (4.42)	158 (3.92)	.25
Current or former	451 920 (95.33)	448 059 (95.33)	3861 (95.78)	
Missing	1185 (0.25)	1173 (0.25)	12 (0.30)	
Tobacco consumption status				
Never	259 450 (54.73)	257 717 (54.83)	1733 (42.99)	<.001
Current or former	212 185 (44.76)	209 910 (44.66)	2275 (56.44)	
Missing	2420 (0.51)	2397 (0.51)	23 (0.57)	
Healthy diet score				
0 to 1	68 305 (14.41)	67 677 (14.40)	628 (15.58)	.06
2 to 3	254 608 (53.71)	252 441 (53.71)	2167 (53.76)	
4 to 5	151 142 (31.88)	149 906 (31.89)	1236 (30.66)	
Physical activity				
Never	30 610 (6.46)	30 255 (6.44)	355 (8.81)	<.001
Low activity	32 112 (6.77)	31 784 (6.76)	328 (8.14)	
Medium activity	346 240 (73.04)	343 448 (73.07)	2792 (69.26)	
High activity	61 885 (13.05)	61 371 (13.06)	514 (12.75)	
Missing	3208 (0.68)	3166 (0.67)	42 (1.04)	
TDI				
<−3.18	156 498 (33.01)	155 262 (33.03)	1236 (30.66)	<.001
−3.18 to −0.64	159 700 (33.69)	158 412 (33.70)	1288 (31.95)	
>−0.64	157 857 (33.30)	156 350 (33.26)	1507 (37.39)	
Kinship to other participants				
No	330 377 (69.69)	327 652 (69.71)	2725 (67.60)	.01
Yes	143 669 (30.31)	142 363 (30.29)	1306 (32.40)	
Missing	9 (0.00)	9 (0.00)	0 (0.00)	
Hypertension	114 465 (24.15)	113 294 (24.10)	1171 (29.05)	<.001
Diabetes	24 921 (5.26)	24 599 (5.23)	322 (7.99)	<.001
Hypercholesterolemia	53 136 (11.21)	52 523 (11.17)	613 (15.21)	<.001
Genetic risk category				
Low	159 758 (33.70)	158 812 (33.79)	946 (23.47)	<.001
Intermediate	159 399 (33.62)	158 124 (33.64)	1275 (31.63)	
High	154 898 (32.68)	153 088 (32.57)	1810 (44.90)	
PM_2.5_				
Q1	118 993 (25.10)	117 932 (25.09)	1061 (26.32)	.04
Q2	118 841 (25.07)	117 876 (25.08)	965 (23.94)	
Q3	118 841 (25.07)	117 790 (25.06)	1051 (26.07)	
Q4	117 380 (24.76)	116 426 (24.77)	954 (23.67)	
PM_10_				
Q1	118 907 (25.08)	117 865 (25.08)	1042 (25.85)	.53
Q2	118 947 (25.09)	117 965 (25.10)	982 (24.36)	
Q3	118 817 (25.06)	117 821 (25.07)	996 (24.71)	
Q4	117 384 (24.76)	116 373 (24.76)	1011 (25.08)	
NO_2_				
Q1	119 072 (25.12)	118 113 (25.13)	959 (23.79)	.02
Q2	119 007 (25.10)	118 040 (25.11)	967 (23.99)	
Q3	118 795 (25.06)	117 727 (25.05)	1068 (26.49)	
Q4	117 181 (24.72)	116 144 (24.71)	1037 (25.73)	
NO_x_				
Q1	118 936 (25.09)	117 975 (25.10)	961 (23.84)	.02
Q2	118 878 (25.08)	117 918 (25.09)	960 (23.82)	
Q3	118 884 (25.08)	117 830 (25.07)	1054 (26.15)	
Q4	117 357 (24.76)	116 301 (24.74)	1056 (26.20)	

Abbreviations: BMI, body mass index (calculated as weight in kilograms divided by height in meters squared); NO_2_, nitrogen dioxide; NO_x_, nitrogen oxides; PM_2.5_, fine particulate matter with a diameter less than 2.5 μm; PM_10_, particulate matter with a diameter less than 10 μm; Q, quartile; TDI, Townsend Deprivation Index.

**Figure 1.**
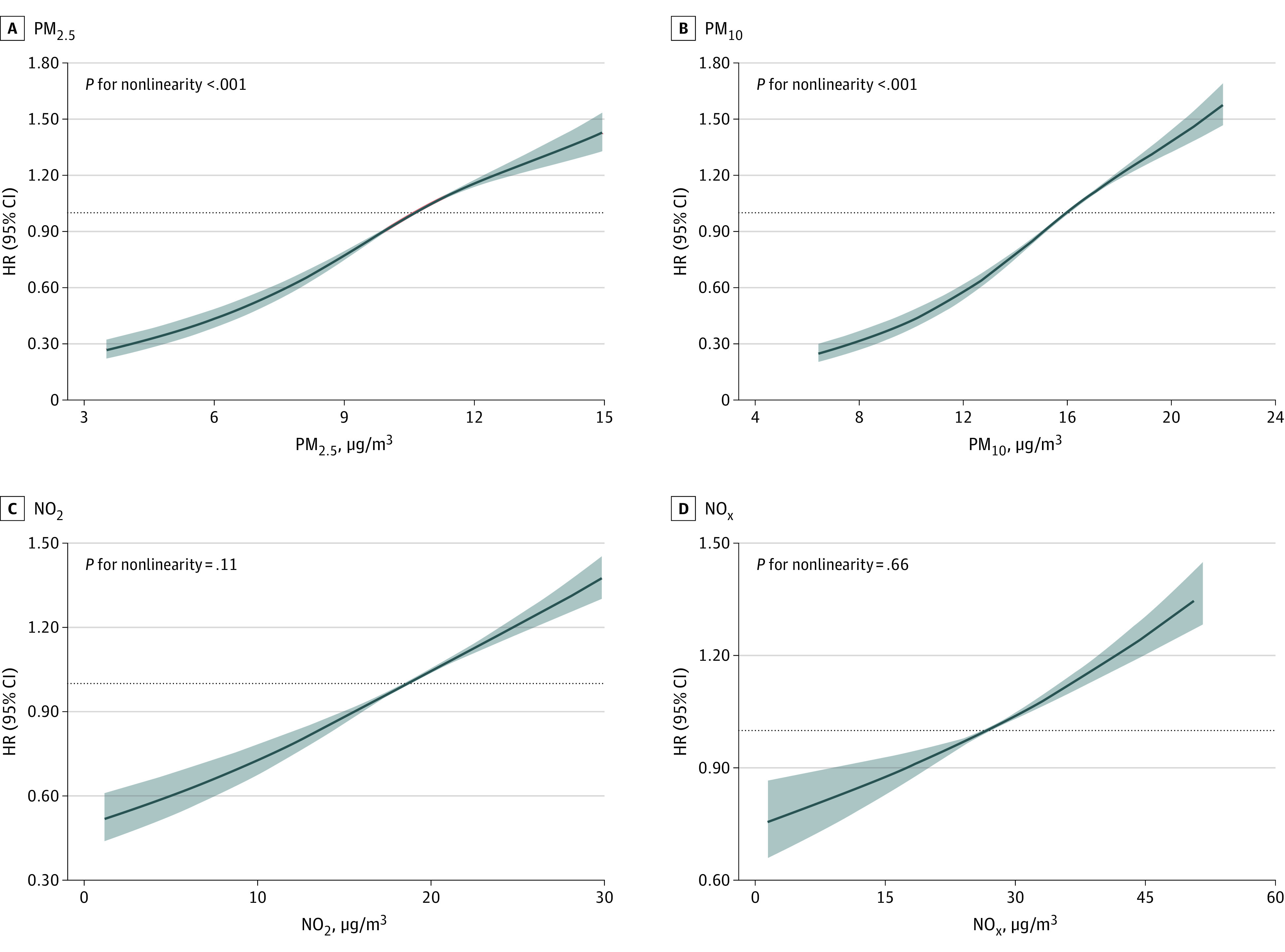
Associations of Long-Term Exposure to Fine Particulate Matter With a Diameter Less Than 2.5 µm (PM_2.5_), Particulate Matter With a Diameter Less Than 10 µm (PM_10_), Nitrogen Dioxide (NO_2_), and Nitrogen Oxides (NO_x_) With the Risk of Psoriasis A restricted cubic spline regression model with 3 knots (at the 10th, 50th, and 90th percentiles) was used to estimate the dose-response associations between air pollutants and the risk of psoriasis among participants. Hazard ratios (HRs, solid lines) and 95% CIs (shaded areas) were adjusted for age, sex, ethnicity, Townsend Deprivation Index, education, employment, alcohol consumption status, tobacco consumption status, healthy diet score, physical activity, body mass index, kinship to other participants, hypertension, diabetes, and hypercholesterolemia.

Table 2 displays the HRs for different quartiles of air pollution exposure and psoriasis risk. Using Cox models analysis, an association was found between all examined air pollutants and an augmented risk of developing psoriasis. The hazard ratios (HRs) per IQR increment for PM_2.5_, PM_10_, NO_2_, and NO_X_ were 1.41 (95% CI, 1.35-1.46), 1.47 (95% CI, 1.41-1.52), 1.28 (95% CI, 1.23-1.33), and 1.19 (95% CI, 1.14-1.24), respectively. Individuals living in areas with the highest quartile of air pollutants (Q4) compared with the lowest quartile (Q1) had multivariable-adjusted HRs of 2.01 (95% CI, 1.83-2.20) for PM_2.5_, 2.21 (95% CI, 2.02-2.43) for PM_10_, 1.64 (95% CI, 1.49-1.80) for NO_2_, and 1.34 (95% CI, 1.22-1.47) for NO_X_ (all *P* < .001). The E-values for HRs of psoriasis were relatively large, implying that the unmeasured confounders in the process of analyses were unlikely to counteract these discovered associations. Figure 1 delineates the dose-response associations between psoriasis incidence and pollutant concentrations of PM_2.5_ (*P* for nonlinearity < .001), PM_10_ (*P* for nonlinearity < .001), NO_2_ (*P* for nonlinearity = .11), and NO_X_ (*P* for nonlinearity = .66). All air pollutants showed a monotonically increasing trend with psoriasis risk. Associations between exposure to air pollutants and psoriasis at low concentrations were also investigated (eTable 2 in Supplement 1). Except for NO_X_ (HR, 1.00; 95% CI, 0.93-1.08), PM_2.5_ (HR, 1.53; 95% CI, 1.42-1.64), PM_10_ (HR, 1.49; 95% CI. 1.41-1.56), and NO_2_ (HR, 1.28; 95% CI, 1.23-1.34) were associated with risk of psoriasis (all *P* < .001).

**Table 2.** Associations Between Air Pollutants and the Risk of Incident Psoriasis

Level of exposure by air pollutant	HR (95% CI)^a^	*P* value	*P* for trend	E-value
PM_2.5_				
Q1, lowest	1 [Reference]	NA	<.001	NA
Q2	1.51 (1.39-1.64)	<.001		2.39
Q3	1.63 (1.49-1.78)	<.001		2.64
Q4, highest	2.01 (1.83-2.20)	<.001		3.43
PM_2.5,_ per IQR increase^b^	1.41 (1.35-1.46)	<.001	NA	2.17
PM_10_				
Q1, lowest	1 [Reference]	NA	<.001	NA
Q2	1.46 (1.34-1.59)	<.001		2.28
Q3	1.70 (1.55-1.85)	<.001		2.79
Q4, highest	2.21 (2.02-2.43)	<.001		3.85
PM_10_, per IQR increase^c^	1.47 (1.41-1.52)	<.001	NA	2.30
NO_2_				
Q1, lowest	1 [Reference]	NA	<.001	NA
Q2	1.14 (1.04-1.24)	.005		1.54
Q3	1.46 (1.33-1.60)	<.001		2.28
Q4, highest	1.64 (1.49-1.80)	<.001		2.66
NO_2,_ per IQR increase^d^	1.28 (1.23-1.33)	<.001	NA	1.88
NO_x_				
Q1, lowest	1 [Reference]	NA	<.001	NA
Q2	0.95 (0.87-1.04)	.24		1.29
Q3	1.16 (1.06-1.27)	<.001		1.59
Q4, highest	1.34 (1.22-1.47)	<.001		2.01
NO_x,_ per IQR increase^e^	1.19 (1.14-1.24)	<.001	NA	1.67

Abbreviations: HR, hazard ratio; NA, not applicable; NO_2_, nitrogen dioxide; NO_x_, nitrogen oxides; PM_2.5_, fine particulate matter with a diameter less than 2.5 μm; PM_10_, particulate matter with a diameter less than 10 μm; Q, quartile;

^a^ Cox regression models adjusted for age, sex, ethnicity, Townsend Deprivation Index, education, employment, alcohol consumption status, tobacco consumption status, healthy diet score, physical activity, body mass index, kinship to other participants, hypertension, diabetes, and hypercholesterolemia.

^b^ One IQR for PM_2.5_ was 2.91 μg/m^3^.

^c^ One IQR for PM_10_ was 3.91 μg/m^3^.

^d^ One IQR for NO_2_ was 7.98 μg/m^3^.

^e^ One IQR for NO_x_ was 14.23 μg/m^3^.

**Figure 2.**
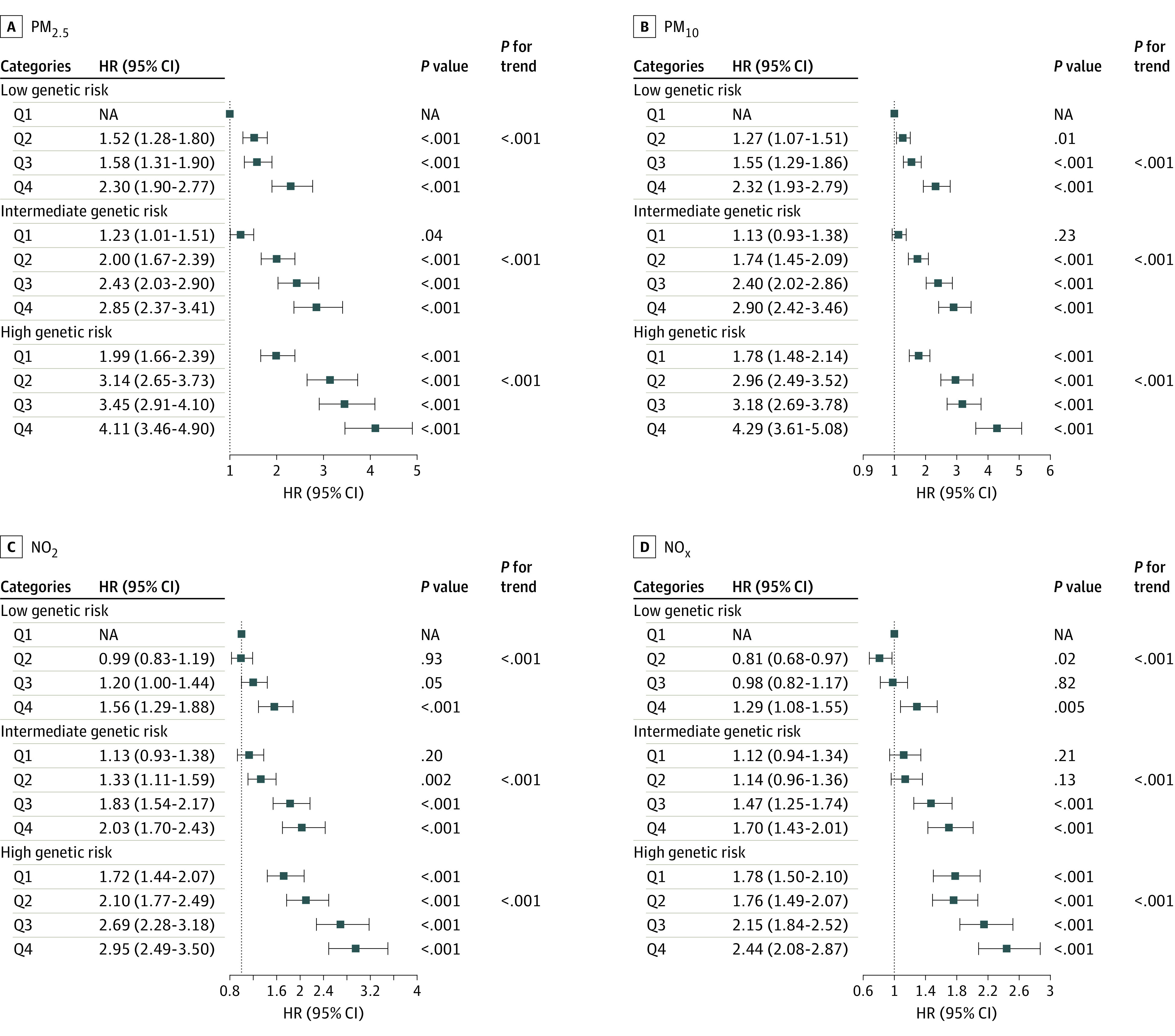
Joint Associations of Long-Term Exposure to Fine Particulate Matter With a Diameter Less Than 2.5 µm (PM_2.5_), Particulate Matter With a Diameter Less Than 10 µm (PM_10_), Nitrogen Dioxide (NO_2_), Nitrogen Oxides (NO_x_), and Polygenetic Risk Score With the Risk of Incident Psoriasis Cox regression models adjusted for age, sex, ethnicity, Townsend Deprivation Index, education, employment, alcohol consumption status, tobacco consumption status, healthy diet score, physical activity, body mass index, kinship to other participants, hypertension, diabetes, hypercholesterolemia, genotyping batch, and the first 10 genetic principal components. HR indicates hazard ratio; NA, not applicable; Q1, lowest quartile of exposure to air pollution; and Q4, highest quartile.

There was an association between the psoriasis PRS and the likelihood of the disease. Participants categorized as having intermediate or high genetic risk had increased risk of psoriasis onset, with HRs of 1.35 (95% CI, 1.24-1.47) and 2.01 (95% CI, 1.85-2.18), respectively, compared with those with low genetic risk (*P* < .001) (eTable 3 in Supplement 1). Concurrently, an assessment of the joint association of air pollution and the PRS with psoriasis susceptibility was conducted (Figure 2). The cumulative association of both genetic risk and air pollution exposure with psoriasis risk appeared in a dose-response framework. The findings indicated the most substantial risk of psoriasis development in participants exposed to elevated air pollution levels concomitant with high genetic risk (PM_2.5_: HR, 4.11; 95% CI, 3.46, 4.90; PM_10_: HR, 4.29; 95% CI, 3.61-5.08; NO_2_: HR, 2.95; 95% CI, 2.49-3.50; NOx: HR, 2.44; 95% CI, 2.08-2.87) compared with those with low exposure and genetic risk. We also observed a multiplicative interaction between PM_10_ and genetic predisposition (*P *for interaction = .002) (eTable 4 in Supplement 1).

A series of sensitivity analyses were conducted to examine the validity of our findings. Sensitivity analyses involved excluding participants diagnosed with psoriasis within the initial 2 years of follow-up (eTable 5 in Supplement 1) and excluding participants with poor self-reported health at baseline; there were no noticeable alterations in the risk ratios associated with psoriasis (eTable 6 in Supplement 1). Our analysis was further refined to only include participants who had resided at their current address for longer than 5 years, revealing no modifications to the results (eTable 7 in Supplement 1). The associations between air pollution and psoriasis remained unchanged after restricting participants to those who did not move during the follow-up period (eTable 8 in Supplement 1). We also performed a sensitivity analysis using the selected covariates determined by the DAG (eFigure in Supplement 1), and the results did not change (eTable 9 in Supplement 1).

## Discussion

In this study, we explored the association between long-term exposure to air pollution and the risk of psoriasis, while also taking the interaction between air pollution and genetics for incident psoriasis into account. By analyzing the data on a national scale, a discernible association was established between long-term exposure to air pollution and an elevated incidence of psoriasis. Additionally, our study elucidated that the interaction between genetic susceptibility and exposure to air pollution contributes to the onset of psoriasis. An enhanced risk of developing psoriasis was identified among participants characterized by elevated levels of air pollution and a high genetic risk. To our knowledge, this investigation is pioneering in providing a comprehensive evaluation at the national level of the association of long-term exposure to air pollution with psoriasis onset. The findings of our research suggest possible avenues for risk assessment and early intervention strategies in high-risk populations that may lead to more effective preventive measures for psoriasis.

The short-term exposure to single air pollutants, such as PM_2.5_, has been confirmed by several studies to be associated with hospital visits for psoriasis.^12,13,14,15^ However, these studies on acute effects often underestimated the disease risk.^35^ Therefore, our study is based on a prospective cohort with a large sample size and rich covariates, providing robust evidence to evaluated long-term exposure to multiple air pollutants and increased risk of psoriasis incidence.

Several studies involving both animal and human participants have examined the biological mechanisms linking air pollutants and the onset of psoriasis.^36^ These investigations posit that air pollution potentially exerts detrimental effects on the skin through various mechanisms, including affecting skin microbial flora, activating aromatic hydrocarbon receptors, inducing inflammatory reactions, and oxidative stress.^37^ These mechanisms may be similar to the pathogenic mechanisms of psoriasis. For example, evidence indicates that polycyclic aromatic hydrocarbons in PM_2.5_ produce adverse effects by activating aromatic hydrocarbon receptors, which may lead to autoimmune diseases.^37^ Transcriptomic analysis shows that PM_2.5_ in the air may affect the expression of genes and pro-inflammatory cytokines related to psoriasis and have adverse effects on human keratinocytes.^38^ Moreover, studies on human embryonic stem cells have reported that PM_2.5_ can disrupt the differentiation of keratinocytes and affect gene expression associated with inflammation and psoriasis.^39^ Overall, there is a need for more comprehensive research to elucidate the mechanisms behind the impact of PM_2.5_ on psoriasis.

While prior investigations have examined the role of genetic predisposition in psoriasis susceptibility, the joint associations of genetic factors and exposure to air pollution with psoriasis remain unexplored. Our findings indicate a positive association between heightened genetic risk, increased exposure to air pollutants, and the subsequent risk of developing psoriasis, displaying a monotonically increasing trend. Furthermore, we identified multiplicative interactions between air pollutants and PRS, attributable to the combination of high pollutant exposure and elevated genetic risk. This interaction surpasses the sum of the individual associations of air pollutants and genetic risk, which has implications for environmental epidemiology and underscores the importance of targeted preventive and health care interventions for populations with heightened genetic risk and substantial exposure to air pollution.

The strength of this study is primarily attributed to the utilization of longitudinal data and genetic information from the UK Biobank, which allows for an assessment of the outcomes of exposure to air pollution on a large-scale and highly representative populations, ensuring both authenticity and statistical robustity.^18^ By using a cohort study design, we were able to exclude individuals with preexisting psoriasis, observe and record the initial onset of psoriasis, and consequently strengthen the argument for a causal connection. Furthermore, this study fills the gap of previous research that mainly focused on the health threats of PM_2.5_ but ignored NO_x_ pollutants for psoriasis. Given the importance of genetic factors in psoriasis, our use of a PRS can quantify genetic risk to help identify high-risk individuals. Our findings underscore the significance of the interaction between air pollution and genetics for incident psoriasis. This has substantial implications for the precision of risk assessments and the timely initiation of preventive measures in susceptible populations. As a chronic disease that currently cannot be cured, our findings can provide evidence for preventive measures.

### Limitations

There are some limitations of this study. First, because the UK Biobank recruited volunteers rapidly and most of participants in the UK Biobank were healthy, selection bias may not be completely avoided. Even though the UK Biobank is a selective group in the UK population, the effect sizes appear to be comparable with those from population-representative samples.^40^ Nevertheless, we know such bias cannot be completely avoided.^41^ Future studies need to focus on this issue. Furthermore, the PRS, constrained by the existing knowledge of single-nucleotide variant loci, does not encapsulate all potential genetic variants that might affect susceptibility to psoriasis. It is conceivable that additional, unidentified genetic variants or mechanisms may contribute to psoriasis risk.^42^ Third, White European individuals make up a large proportion of participants in the UK Biobank, which hinders our ability to gain insights into the genetic risk of different populations. In the future, the results of this study need to be verified in diverse populations. Fourth, it is worth noting that results reported from observational study designs are often susceptible to confounding or other forms of bias.^43^ Like other large health databases, the UK Biobank does not provide data on indoor air pollution, exposure at the workplace or during commuting, and other relevant individual behavior data beyond smoking. More studies are needed for further investigation. Fifth, causal inferences cannot be obtained in a cohort study, and the results should be interpreted with caution. Future randomized clinical trials are needed for further verification. Moreover, there is a potential gap between annual exposure and actual exposure, especially in scenarios where an individual encounters censoring events early in the year. Despite the consistent temporal patterns of air pollutant concentrations observed throughout the study period in the UK, annual exposure can somewhat serve as a proxy for real-time exposure.^44^ To address these limitations, future research should strive to incorporate more detailed individual-level exposure assessments.

## Conclusions

In summary, this investigation used prospective data to explore the associations of various air pollutants with the development of psoriasis. The data revealed an increase in psoriasis risk among participants with higher genetic vulnerability and increased exposure to air pollutants. Consequently, there is a need to devise and implement effective interventions aimed at mitigating air pollution and safeguarding individuals from the effects associated with psoriasis.

## Notes

Supplement 1. **eTable 1.** Range of Air Pollutant Concentrations in the UK Biobank **eTable 2.** Associations Between Air Pollutants in Low-Level Concentrations and the Risk of Incident Psoriasis **eTable 3.** Associations of Polygenic Risk Score With the Risk of Incident Psoriasis **eTable 4.** Additive and Multiplicative Interactions Between Air Pollutants and Polygenic Risk Score for the Risk of Incident Psoriasis **eTable 5.** Associations Between Air Pollutants and the Risk of Incident Psoriasis After Excluding Participants Diagnosed With Psoriasis During the First 2 Years of Follow-Up **eTable 6.** Associations Between Air Pollutants and the Risk of Incident Psoriasis Among Participants Without Poor Self-Reported Health at Baseline **eTable 7.** Associations Between Air Pollutants and the Risk of Incident Psoriasis Among Participants With Length of Living Time at Current Address of More Than 5 Years **eTable 8.** Associations Between Air Pollutants and the Risk of Incident Psoriasis Among Participants Without Home Address Change During Follow-Up **eTable 9.** Associations Between Air Pollutants and the Risk of Incident Psoriasis After Filtering Covariates by Directed Acyclic Graph **eFigure.** Directed Acyclic Graph for the Associations Between Air Pollutants and Psoriasis

Supplement 2.
Data Sharing Statement
