# Supplementary material for: Exposure to Air Pollution, Genetic Susceptibility, and Psoriasis Risk in the UK
Source: JAMA Netw Open. 2024 Jul 16;7(7):e2421665. doi: 10.1001/jamanetworkopen.2024.21665 (PMC11252902; doi:10.1001/jamanetworkopen.2024.21665)
Supplement: Supplement 1. — eTable 1. Range of Air Pollutant Concentrations in the UK Biobank eTable 2. Associations Between Air Pollutants in Low-Level Concentrations and the Risk of Incident Psoriasis eTable 3. Associations of Polygenic Risk Score With the Risk of Incident Psoriasis eTable 4. Additive and Multiplicative Interactions Between Air Pollutants and Polygenic Risk Score for the Risk of Incident Psoriasis eTable 5. Associations Between Air Pollutants and the Risk of Incident Psoriasis After Excluding Participants Diagnosed With Psoriasis During the First 2 Years of Follow-Up eTable 6. Associations Between Air Pollutants and the Risk of Incident Psoriasis Among Participants Without Poor Self-Reported Health at Baseline eTable 7. Associations Between Air Pollutants and the Risk of Incident Psoriasis Among Participants With Length of Living Time at Current Address of More Than 5 Years eTable 8. Associations Between Air Pollutants and the Risk of Incident Psoriasis Among Participants Without Home Address Change During Follow-Up eTable 9. Associations Between Air Pollutants and the Risk of Incident Psoriasis After Filtering Covariates by Directed Acyclic Graph eFigure. Directed Acyclic Graph for the Associations Between Air Pollutants and Psoriasis [file jamanetwopen-e2421665-s001.pdf]

Supplemental Online Content

Wu J, Ma Y, Yang J, Tian Y. Exposure to air pollution, genetic susceptibility, and psoriasis risk in the UK. *JAMA Netw Open.* 2024;7(7):e2421665. doi:10.1001/jamanetworkopen.2024.21665

eTable 1. Range of Air Pollutant Concentrations in the UK Biobank

eTable 2. Associations Between Air Pollutants in Low-Level Concentrations and the Risk of Incident Psoriasis

eTable 3. Associations of Polygenic Risk Score With the Risk of Incident Psoriasis

eTable 4. Additive and Multiplicative Interactions Between Air Pollutants and Polygenic Risk Score for the Risk of Incident Psoriasis

eTable 5. Associations Between Air Pollutants and the Risk of Incident Psoriasis After Excluding Participants Diagnosed With Psoriasis During the First 2 Years of Follow-Up

eTable 6. Associations Between Air Pollutants and the Risk of Incident Psoriasis Among Participants Without Poor Self-Reported Health at Baseline

eTable 7. Associations Between Air Pollutants and the Risk of Incident Psoriasis Among Participants With Length of Living Time at Current Address of More Than 5 Years

eTable 8. Associations Between Air Pollutants and the Risk of Incident Psoriasis Among Participants Without Home Address Change During Follow-Up

eTable 9. Associations Between Air Pollutants and the Risk of Incident Psoriasis After Filtering Covariates by Directed Acyclic Graph

eFigure. Directed Acyclic Graph for the Associations Between Air Pollutants and Psoriasis

This supplemental material has been provided by the authors to give readers additional information about their work.

© 2024 Wu J et al. *JAMA Network Open.*

eTable 1. Range of Air Pollutant Concentrations in the UK Biobank

Air pollutant	Mean (SD), μg/m^3	Q1, μg/m^3	Q2, μg/m^3	Q3, μg/m^3	Q4, μg/m^3
$PM_{2.5}$	10.66 (2.27)	<9.11	9.11-10.62	10.62-12.02	⩾12.02
PM_{10}	16.08 (3.06)	<13.98	13.98-15.98	15.98-17.89	⩾17.89
NO_2	19.11 (6.46)	<14.60	14.60-18.61	18.61-22.58	⩾22.58
NO_x	28.71 (12.11)	<20.25	20.25-26.77	26.77-34.49	⩾34.49

© 2024 Wu J et al. *JAMA Network Open.*

eTable 2. Associations Between Air Pollutants in Low-Level Concentrations and the Risk of Incident Psoriasis

Air pollutant	HR (95% CI)	*P* value	*P* for trend
$PM_{2.5}$			
Q1	Ref.	--	
Q2	**1.61 (1.41, 1.84)**	**<0.001**	**<0.001**
Q3	**1.79 (1.55, 2.06)**	**<0.001**	
Q4	**1.99 (1.73, 2.29)**	**<0.001**	
$PM_{2.5}$, per IQR increase	**1.53 (1.42, 1.64)**	**<0.001**	**--**
PM_{10}			
Q1	Ref.	--	
Q2	**1.49 (1.36, 1.63)**	**<0.001**	**<0.001**
Q3	**1.66 (1.51, 1.82)**	**<0.001**	
Q4	**2.03 (1.84, 2.23)**	**<0.001**	
PM_{10}, per IQR increase	**1.49 (1.41, 1.56)**	**<0.001**	**--**
NO_2			
Q1	Ref.	--	
Q2	**1.13 (1.04, 1.24)**	**0.007**	**<0.001**
Q3	**1.46 (1.33, 1.59)**	**<0.001**	
Q4	**1.60 (1.45, 1.76)**	**<0.001**	
NO_2, per IQR increase	**1.28 (1.23, 1.34)**	**<0.001**	**--**
NO_x			
Q1	Ref.	--	
Q2	1.06 (0.94, 1.20)	0.341	0.182
Q3	0.92 (0.81, 1.05)	0.216	
Q4	1.02 (0.90, 1.16)	0.776	
NO_x, per IQR increase	1.00 (0.93, 1.08)	0.952	**--**

Cox regression models adjusted for age, sex, ethnicity, TDI, education, employment, alcohol consumption status, tobacco consumption status, healthy diet score, physical activity, BMI, kinship to other participants, hypertension, diabetes, and hypercholesterolemia.

© 2024 Wu J et al. *JAMA Network Open.*

eTable 3. Associations of Polygenic Risk Score With the Risk of Incident Psoriasis

PRS	HR (95% CI)	*P* value	*P* for trend
PRS categories			
Low genetic risk	Ref.	--	
Intermediate genetic risk	**1.35 (1.24, 1.47)**	**<0.001**	**<0.001**
High genetic risk	**2.01 (1.85, 2.18)**	**<0.001**	
PRS, per IQR[a] increase	**1.39 (1.35, 1.44)**	**<0.001**	--

[a] IQR of PRS was 1.13.

Cox regression models adjusted for age, sex, ethnicity, TDI, education, employment, alcohol consumption status, tobacco consumption status, healthy diet score, physical activity, BMI, kinship to other participants, hypertension, diabetes, hypercholesterolemia, genotyping batch, and the first ten genetic principal components.

© 2024 Wu J et al. *JAMA Network Open.*

eTable 4. Additive and Multiplicative Interactions Between Air Pollutants and Polygenic Risk Score for the Risk of Incident Psoriasis

	RERI (95% CI)	AP (95% CI)	*P* for interaction
**$PM_{2.5}$**			
**Intermediate genetic risk**			
Q2	0.25 (-0.07, 0.56)	0.12 (-0.04, 0.28)	
Q3	**0.62 (0.33, 0.89)**	**0.25 (0.13, 0.37)**	
Q4	0.32 (-0.00, 0.68)	0.11 (-0.00, 0.24)	0.105
**High genetic risk**			
Q2	**0.64 (0.34, 0.93)**	**0.20 (0.11, 0.30)**	
Q3	**0.88 (0.51, 1.20)**	**0.26 (0.15, 0.34)**	
Q4	**0.83 (0.47, 1.22)**	**0.20 (0.12, 0.29)**	
**PM_{10}**			
**Intermediate genetic risk**			
Q2	**0.34 (0.03, 0.61)**	**0.19 (0.02, 0.35)**	
Q3	**0.72 (0.43, 1.00)**	**0.30 (0.18, 0.41)**	
Q4	**0.45 (0.14, 0.79)**	**0.15 (0.05, 0.27)**	**0.002**
**High genetic risk**			
Q2	**0.91 (0.59, 1.20)**	**0.31 (0.20, 0.40)**	
Q3	**0.86 (0.55, 1.12)**	**0.27 (0.17, 0.35)**	
Q4	**1.19 (0.84, 1.55)**	**0.28 (0.20, 0.35)**	
**NO_2**			
**Intermediate genetic risk**			
Q2	0.20 (-0.04, 0.42)	0.15 (-0.03, 0.31)	
Q3	**0.49 (0.27, 0.74)**	**0.27 (0.15, 0.39)**	
Q4	**0.34 (0.12, 0.58)**	**0.17 (0.06, 0.28)**	0.051
**High genetic risk**			
Q2	**0.38 (0.13, 0.65)**	**0.18 (0.06, 0.31)**	
Q3	**0.77 (0.49, 1.05)**	**0.28 (0.19, 0.38)**	
Q4	**0.67 (0.35, 1.01)**	**0.23 (0.12, 0.33)**	
**NO_x**			
**Intermediate genetic risk**			0.053

© 2024 Wu J et al. *JAMA Network Open.*

Q2	**0.21 (0.02, 0.40)**	**0.19 (0.02, 0.35)**
Q3	**0.37 (0.18, 0.58)**	**0.25 (0.13, 0.38)**
Q4	**0.28 (0.10, 0.48)**	**0.17 (0.06, 0.28)**
**High genetic risk**		
Q2	0.17 (-0.07, 0.44)	0.10 (-0.04, 0.25)
Q3	**0.40 (0.14, 0.66)**	**0.18 (0.07, 0.30)**
Q4	**0.37 (0.10, 0.67)**	**0.15 (0.04, 0.27)**

Cox regression models adjusted for age, sex, ethnicity, TDI, education, employment, alcohol consumption status, tobacco consumption status, healthy diet score, physical activity, BMI, kinship to other participants, hypertension, diabetes, hypercholesterolemia, genotyping batch, and the first ten genetic principal components.

© 2024 Wu J et al. *JAMA Network Open.*

eTable 5. Associations Between Air Pollutants and the Risk of Incident Psoriasis After Excluding Participants Diagnosed With Psoriasis During the First 2 Years of Follow-Up

Air pollutant	HR (95% CI)	*P* value	*P* for trend
$PM_{2.5}$			
Q1	Ref.	--	
Q2	**1.52 (1.39, 1.67)**	**<0.001**	**<0.001**
Q3	**1.63 (1.48, 1.79)**	**<0.001**	
Q4	**2.05 (1.85, 2.27)**	**<0.001**	
$PM_{2.5}$, per IQR increase	**1.42 (1.36, 1.48)**	**<0.001**	**--**
PM_{10}			
Q1	Ref.	--	
Q2	**1.49 (1.36, 1.63)**	**<0.001**	**<0.001**
Q3	**1.66 (1.51, 1.83)**	**<0.001**	
Q4	**2.20 (1.99, 2.43)**	**<0.001**	
PM_{10}, per IQR increase	**1.45 (1.39, 1.52)**	**<0.001**	**--**
NO_2			
Q1	Ref.	--	
Q2	**1.11 (1.00, 1.22)**	**0.043**	**<0.001**
Q3	**1.42 (1.29, 1.57)**	**<0.001**	
Q4	**1.59 (1.43, 1.77)**	**<0.001**	
NO_2, per IQR increase	**1.27 (1.21, 1.33)**	**<0.001**	**--**
NO_x			
Q1	Ref.	--	
Q2	0.96 (0.88, 1.06)	0.444	**<0.001**
Q3	**1.17 (1.06, 1.29)**	**0.001**	
Q4	**1.33 (1.20, 1.47)**	**<0.001**	
NO_x, per IQR increase	**1.18 (1.13, 1.23)**	**<0.001**	**--**

Cox regression models adjusted for age, sex, ethnicity, TDI, education, employment, alcohol consumption status, tobacco consumption status, healthy diet score, physical activity, BMI, kinship to other participants, hypertension, diabetes, and hypercholesterolemia.

© 2024 Wu J et al. *JAMA Network Open.*

eTable 6. Associations Between Air Pollutants and the Risk of Incident Psoriasis Among Participants Without Poor Self-Reported Health at Baseline

Air pollutant	HR (95% CI)	*P* value	*P* for trend
$PM_{2.5}$			
Q1	Ref.	--	
Q2	**1.54 (1.42, 1.68)**	**<0.001**	**<0.001**
Q3	**1.67 (1.52, 1.84)**	**<0.001**	
Q4	**2.05 (1.86, 2.26)**	**<0.001**	
$PM_{2.5}$, per IQR increase	**1.43 (1.37, 1.49)**	**<0.001**	**--**
PM_{10}			
Q1	Ref.	--	
Q2	**1.46 (1.34, 1.60)**	**<0.001**	**<0.001**
Q3	**1.72 (1.56, 1.88)**	**<0.001**	
Q4	**2.28 (2.07, 2.51)**	**<0.001**	
PM_{10}, per IQR increase	**1.48 (1.42, 1.55)**	**<0.001**	**--**
NO_2			
Q1	Ref.	--	
Q2	**1.11 (1.01, 1.22)**	**0.032**	**<0.001**
Q3	**1.48 (1.34, 1.63)**	**<0.001**	
Q4	**1.63 (1.48, 1.80)**	**<0.001**	
NO_2, per IQR increase	**1.29 (1.23, 1.34)**	**<0.001**	**--**
NO_x			
Q1	Ref.	--	
Q2	**0.94 (0.86, 1.03)**	**0.176**	**<0.001**
Q3	**1.17 (1.07, 1.28)**	**<0.001**	
Q4	**1.36 (1.24, 1.50)**	**<0.001**	
NO_x, per IQR increase	**1.20 (1.15, 1.25)**	**<0.001**	**--**

Cox regression models adjusted for age, sex, ethnicity, TDI, education, employment, alcohol consumption status, tobacco consumption status, healthy diet score, physical activity, BMI, kinship to other participants, hypertension, diabetes, and hypercholesterolemia.

© 2024 Wu J et al. *JAMA Network Open.*

eTable 7. Associations Between Air Pollutants and the Risk of Incident Psoriasis Among Participants With Length of Living Time at Current Address of More Than 5 Years

Air pollutant	HR (95% CI)	*P* value	*P* for trend
$PM_{2.5}$			
Q1	Ref.	--	
Q2	**1.49 (1.36, 1.63)**	**<0.001**	**<0.001**
Q3	**1.62 (1.47, 1.78)**	**<0.001**	
Q4	**1.98 (1.79, 2.19)**	**<0.001**	
$PM_{2.5}$, per IQR increase	**1.40 (1.34, 1.47)**	**<0.001**	**--**
PM_{10}			
Q1	Ref.	--	
Q2	**1.43 (1.30, 1.56)**	**<0.001**	**<0.001**
Q3	**1.67 (1.51, 1.84)**	**<0.001**	
Q4	**2.21 (2.00, 2.45)**	**<0.001**	
PM_{10}, per IQR increase	**1.46 (1.40, 1.53)**	**<0.001**	**--**
NO_2			
Q1	Ref.	--	
Q2	**1.11 (1.01, 1.23)**	**0.039**	**<0.001**
Q3	**1.46 (1.32, 1.61)**	**<0.001**	
Q4	**1.64 (1.48, 1.82)**	**<0.001**	
NO_2, per IQR increase	**1.28 (1.23, 1.34)**	**<0.001**	**--**
NO_x			
Q1	Ref.	--	
Q2	0.92 (0.83, 1.01)	0.077	**<0.001**
Q3	**1.16 (1.05, 1.28)**	**0.003**	
Q4	**1.34 (1.21, 1.49)**	**<0.001**	
NO_x, per IQR increase	**1.19 (1.14, 1.24)**	**<0.001**	**--**

Cox regression models adjusted for age, sex, ethnicity, TDI, education, employment, alcohol consumption status, tobacco consumption status, healthy diet score, physical activity, BMI, kinship to other participants, hypertension, diabetes, and hypercholesterolemia.

© 2024 Wu J et al. *JAMA Network Open.*

eTable 8. Associations Between Air Pollutants and the Risk of Incident Psoriasis Among Participants Without Home Address Change During Follow-Up

Air pollutant	HR (95% CI)	*P* value	*P* for trend	E-value
$PM_{2.5}$				
Q1	Ref.	--		--
Q2	**1.45 (1.32, 1.60)**	**<0.001**	**<0.001**	2.26
Q3	**1.59 (1.43, 1.77)**	**<0.001**		2.56
Q4	**1.99 (1.78, 2.22)**	**<0.001**		3.39
$PM_{2.5}$, per IQR increase	**1.44 (1.38, 1.52)**	**<0.001**	**--**	2.24
PM_{10}				
Q1	Ref.	--		--
Q2	**1.46 (1.32, 1.61)**	**<0.001**	**<0.001**	2.28
Q3	**1.67 (1.50, 1.86)**	**<0.001**		2.73
Q4	**2.25 (2.02, 2.51)**	**<0.001**		3.93
PM_{10}, per IQR increase	**1.50 (1.43, 1.57)**	**<0.001**	**--**	2.37
NO_2				
Q1	Ref.	--		--
Q2	1.11 (1.00, 1.24)	0.053	**<0.001**	1.46
Q3	**1.50 (1.34, 1.67)**	**<0.001**		2.37
Q4	**1.69 (1.50, 1.89)**	**<0.001**		2.77
NO_2, per IQR increase	**1.32 (1.26, 1.39)**	**<0.001**	**--**	1.97
NO_x				
Q1	Ref.	--		--
Q2	0.94 (0.84, 1.04)	0.229	**<0.001**	1.32
Q3	**1.16 (1.04, 1.29)**	**0.008**		1.59
Q4	**1.40 (1.26, 1.57)**	**<0.001**		2.15
NO_x, per IQR increase	**1.23 (1.17, 1.28)**	**<0.001**	**--**	1.76

Cox regression models adjusted for age, sex, ethnicity, TDI, education, employment, alcohol consumption status, tobacco consumption status, healthy diet score, physical activity, BMI, kinship to other participants, hypertension, diabetes, and hypercholesterolemia.

© 2024 Wu J et al. *JAMA Network Open.*

eTable 9. Associations Between Air Pollutants and the Risk of Incident Psoriasis After Filtering Covariates by Directed Acyclic Graph

Air pollutant	HR (95% CI)	*P* value	*P* for trend	E-value
$PM_{2.5}$				
Q1	Ref.	--		--
Q2	**1.51 (1.39, 1.64)**	**<0.001**	**<0.001**	2.39
Q3	**1.64 (1.50, 1.79)**	**<0.001**		2.66
Q4	**2.02 (1.84, 2.21)**	**<0.001**		3.46
$PM_{2.5}$, per IQR increase	**1.41 (1.36, 1.47)**	**<0.001**	**--**	2.17
PM_{10}				
Q1	Ref.	--		--
Q2	**1.46 (1.34, 1.59)**	**<0.001**	**<0.001**	2.28
Q3	**1.71 (1.56, 1.86)**	**<0.001**		2.81
Q4	**2.24 (2.04, 2.45)**	**<0.001**		3.91
PM_{10}, per IQR increase	**1.47 (1.41, 1.53)**	**<0.001**	**--**	2.30
NO_2				
Q1	Ref.	--		--
Q2	**1.15 (1.05, 1.25)**	**0.003**	**<0.001**	1.57
Q3	**1.47 (1.34, 1.61)**	**<0.001**		2.30
Q4	**1.66 (1.51, 1.83)**	**<0.001**		2.71
NO_2, per IQR increase	**1.28 (1.23, 1.34)**	**<0.001**	**--**	1.88
NO_x				
Q1	Ref.	--		--
Q2	0.95 (0.87, 1.04)	0.306	**<0.001**	1.29
Q3	**1.17 (1.07, 1.28)**	**<0.001**		1.62
Q4	**1.36 (1.24, 1.50)**	**<0.001**		2.06
NO_x, per IQR increase	**1.19 (1.15, 1.24)**	**<0.001**	**--**	1.67

Cox regression models adjusted for age, ethnicity, TDI, education, employment, and tobacco consumption status.

© 2024 Wu J et al. *JAMA Network Open.*

eFigure 1. Directed Acyclic Graph for the Associations Between Air Pollutants and Psoriasis

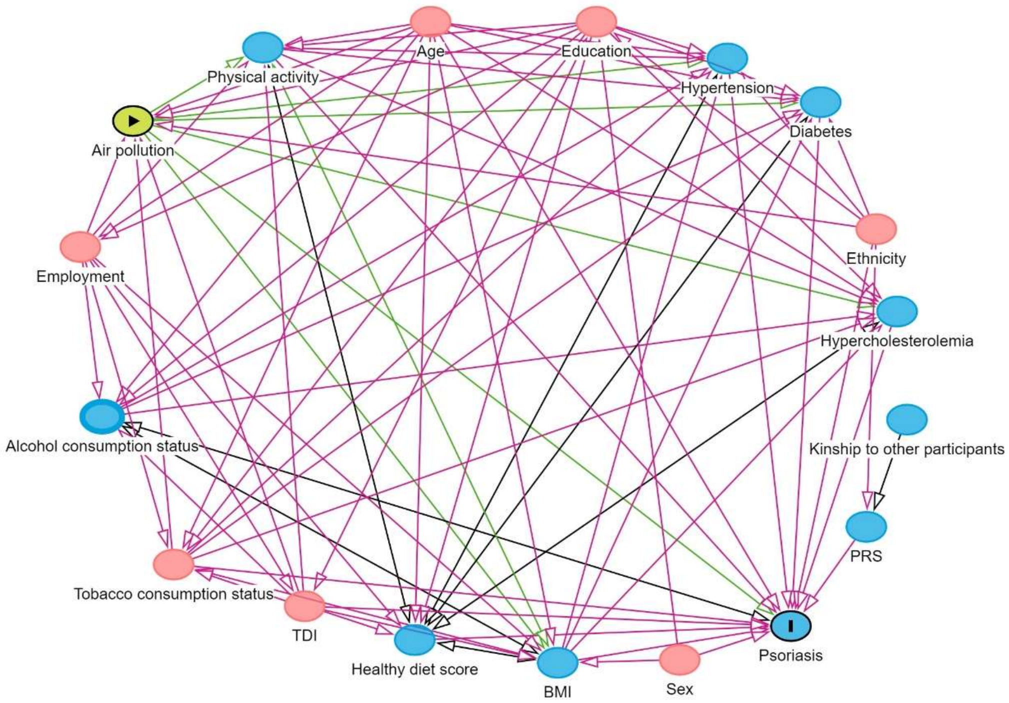

© 2024 Wu J et al. *JAMA Network Open.*
