# Supplementary material for: Exposure to Air Pollution, Genetic Susceptibility, and Psoriasis Risk in the UK
Source: JAMA Netw Open. 2024 Jul 16;7(7):e2421665. doi: 10.1001/jamanetworkopen.2024.21665 (PMC11252902; doi:10.1001/jamanetworkopen.2024.21665)
Supplement: Supplement 2. — Data Sharing Statement [file jamanetwopen-e2421665-s002.pdf]

Data Sharing Statement

Wu. Exposure to Air Pollution, Genetic Susceptibility, and Psoriasis Risk in the UK. *JAMA Netw Open*. Published July 16, 2024. doi:10.1001/jamanetworkopen.2024.21665

Data
Data available: No

Additional Information
Explanation for why data not available: The data utilized in this research can be accessible through the UK Biobank. Accessing the UK Biobank necessitates obtaining permissions, which involves a registration and application procedure. Further details can be located on the website (https://www.ukbiobank.ac.uk/).
